# Cord Blood-Derived Natural Killer Cell Exploitation in Immunotherapy Protocols: More Than a Promise?

**DOI:** 10.3390/cancers14184439

**Published:** 2022-09-13

**Authors:** Laura Damele, Grazia Maria Spaggiari, Monica Parodi, Maria Cristina Mingari, Massimo Vitale, Chiara Vitale

**Affiliations:** 1 Istituto di Ricovero e Cura a Carattere Scientifico (IRCCS) Ospedale Policlinico San Martino, 16132 Genova, Italy; 2 Istituto di Ricovero e Cura a Carattere Scientifico (IRCCS) Istituto Giannina Gaslini, 16147 Genova, Italy; 3 Dipartimento di Medicina Sperimentale, Università di Genova, 16132 Genova, Italy

**Keywords:** NK cell anti-tumor activity, UCB-NK cells, NK cell-based cancer immunotherapy

## Abstract

**Simple Summary:**

NK cell anti-tumor activity against hematological malignancies is well-established and many studies support their role in the control of solid tumor growth and metastasis generation. However, tumor microenvironment may affect NK cell function. Ongoing studies are aimed to design novel immunotherapeutic protocols to combine NK cell-based immunotherapy with other therapeutic strategies to improve the anti-tumor NK cell response. In this context, UCB is one of the main sources of both mature NK cells and of CD34^+^ HSPC that can generate NK cells, both in-vivo and in-vitro. UCB-derived NK cells represent a valuable tool to perform in-vitro and preclinical analyses and are already used in several clinical settings, particularly against hematological malignancies. The present review describes the characteristics of different types of UCB-derived NK cells and the in-vitro models to expand them, both for research and clinical purposes in the context of cancer immunotherapy.

**Abstract:**

In the last 20 years, Natural Killer (NK) cell-based immunotherapy has become a promising approach to target various types of cancer. Indeed, NK cells play a pivotal role in the first-line defense against tumors through major histocompatibility complex-independent immunosurveillance. Their role in the control of leukemia relapse has been clearly established and, moreover, the presence of NK cells in the tumor microenvironment (TME) generally correlates with good prognosis. However, it has also been observed that, often, NK cells poorly infiltrate the tumor tissue, and, in TME, their functions may be compromised by immunosuppressive factors that contribute to the failure of anti-cancer immune response. Currently, studies are focused on the design of effective strategies to expand NK cells and enhance their cytotoxic activity, exploiting different cell sources, such as peripheral blood (PB), umbilical cord blood (UCB) and NK cell lines. Among them, UCB represents an important source of mature NK cells and CD34^+^ Hematopoietic Stem and Progenitor Cells (HSPCs), as precursors of NK cells. In this review, we summarize the UCB-derived NK cell activity in the tumor context, review the different in-vitro models to expand NK cells from UCB, and discuss the importance of their exploitation in anti-tumor immunotherapy protocols.

## 1. Introduction

In recent decades, the exploitation of anti-tumor immune response has become a feasible approach to fight cancer [1,2]. In particular, the advances in the field of in-vivo lymphocyte activation, and of ex-vivo lymphocyte activation, expansion and genetic manipulation (i.e., by the generation of chimeric antigen receptors, CAR), have increased the efficacy of adoptive cellular immunotherapy, leading to a significant improvement of cancer patients’ prognosis [3]. Thus far, NK-cell based immunotherapy has not reached a full success as adoptive T cell therapy, however, preclinical models and ongoing clinical trials suggest that NK cells could play an important role in cancer treatment [4]. In particular, their involvement in the control of hematological malignancies in allogeneic hematopoietic stem cell transplantation (HSCT) has recently pushed the efforts to exploit their potential anti-tumor activity in other types of cancer patients [4,5]. The source of NK cells for therapeutic purposes can be represented by mature NK cells derived from either PB or UCB, but also by NK cells generated from CD34^+^ HSPCs or from induced-pluripotent stem cells (iPSC) [6]. In particular, UCB may represent a feasible source of NK cells, as the worldwide distribution of Cord Blood Banks allows the storage of great amounts of frozen and easily available UCB sample units. In this context, the most important Cord Blood Banks are mainly institutional organizations whose activity is under continuous evaluation and update to create a world-wide network with high-quality standardized protocols of sample collection, storage and exploitation [7].UCB is a suitable source of hematopoietic stem cells (HSCs) for HSCT that leads to a fast in-vivo generation and recovery of NK cells but it may also represent an important source for the “off the shelf” preparation of both mature and HSPC-derived NK cells, which can be further manipulated to improve their functional properties [8,9]. The different methods that have been designed to expand and prepare effective immunotherapeutic NK cell effectors also contributed to improving the knowledge of NK cell biology and development, and represented a powerful tool to test the anti-tumor activity of UCB-derived NK cells in preclinical models. Currently, several encouraging clinical trials are ongoing. The present review will provide an overview of the features of NK cells and of their ability to control tumors, focusing on UCB-NK cell properties. In particular, we will discuss the features of UCB mature NK cells and UCB-HSPC- or UCB-iPSC-derived NK cells and about their potential exploitation for the design of novel immunotherapy protocols.

## 2. Natural Killer Cell Biology

Human NK cells belong to the innate lymphoid cell (ILC) family and are characterized by prominent effector functions. They control viral infections, in particular those caused by herpesviruses (but also recognize Ags from a variety of other viruses) and can maintain a sort of memory through the expansion and persistence of specific NK cell subsets. NK cells also play a pivotal role to prevent tumor growth and progression. In this context, they have been shown to be very important in the control of leukemic relapse. They can exert their effects by cytolytic killing of virally-infected and tumor cells that modulate class I Human Leucocyte Antigens (HLA-I)-molecules. Moreover, they can release inflammatory cytokines, interact with dendritic cells (DC) (influencing their maturation) and, ultimately, regulate the T cell response. In this paragraph, we will summarize the mechanisms underlying their activation and development and their role in the control of solid tumors [10,11].

### 2.1. NK Cell Activation and Development

NK cells represent 5–15% of lymphocytes in PB and originate primarily in the bone marrow (BM) from CD34^+^ HSCs, but they can also develop and be detected in different districts such as the spleen, liver, thymus, secondary lymphoid organs, gut, tonsils and uterus. According to their tissue distribution, they can also show different phenotypic and functional features [12,13,14]. Based on the differential expression of the CD56 and CD16 molecules, NK cells are generally classified into two main subsets: the CD56^bright^CD16^−^ regulatory NK cells and CD56^dim^CD16^+^ mature, cytotoxic NK cells. The CD56^bright^ NK cell subset represents less than 10% of PB-NK cells whereas it covers the majority of NK cells present in peripheral tissues. CD56^bright^ NK cells are poorly cytotoxic but are able to produce several cytokines, including IFN-γ, which promote Th1 CD4^+^ lymphocyte polarization and stimulate DC maturation [10,13,15,16]. Conversely, the CD56^dim^ cell subset covers 90% of circulating NK cells, whereas it is less represented in the tissues. CD56^dim^ NK cells can mediate cytolytic activity through the release of lytic granules containing perforin and granzymes. Antibody-dependent cell cytotoxicity (ADCC) is one of the most important killing mechanisms of CD56^dim^ NK cells that involves the CD16 molecule without the need for any additional costimulatory signals; the recognition of the Fc portion of IgG antibodies bound to target cells promotes NK cell degranulation. Importantly, CD56^dim^ NK cells are also able to produce large amounts of cytokines once activated [15,17,18,19].

NK cell activation depends on the balance between stimulatory and inhibitory signals delivered by receptors that recognize ligands and/or soluble ligands that can be expressed by tumor cells. The main stimulatory receptors, beyond CD16, are represented by the three Natural Cytotoxicity Receptors (NCRs) [20]: NKp46 (CD335), NKp44 (CD336, expressed upon activation) and NKp30 (CD337). Other stimulatory receptors used by NK cells are DNAM-1 (CD226), 2B4(CD244), and some C-type lectins, including NKG2D (CD314), NKp80, and the HLA-E-specific low affinity receptor, NKG2C/CD94 heterodimer [21]. Activating receptor ligands are not yet fully characterized but, generally, they are represented by molecules expressed by cells under stressing and inflammatory conditions, such as viral infections or neoplastic transformations [20,22].

The inhibitory counterpart is mainly represented by the heterodimer CD94/NKG2A (CD159a), recognizing HLA-E molecules and the Killer-Cell Immunoglobulin-like receptors (KIRs, CD158-a/b/e/k), each specific for a different group of classical HLA-I molecules [23,24]. Other important inhibitory receptors include Siglec-7 (CD328), IRP60 (CD300a) and LIR-1 [25]. Finally, the function of NK cells is also under the control of the so-called inhibitory immune checkpoint receptors, such as PD1 (CD279), TIM-3 (CD366) and TIGIT, which can be overexpressed or de-novo induced upon prolonged stimulation within the TME, favoring tumor escape [26,27,28,29].

NK cells originate primarily in the BM from CD34^+^ HSCs, which express the DNA-binding protein inhibitor (ID2) transcriptional regulator. The CD34^+^ cell precursors can also generate other ILCs which display a preferential tissue distribution. Three main groups are well-described on the basis of expressed transcription factors (TF) and cytokine production: ILC1 (T-bet; IFN-γ), ILC2 (GATA-3 and Retinoic Acid Receptor-related Orphan Receptor alfa, RORα; IL-5 and IL-13) and ILC3 (Retinoic Acid Receptor-related Orphan Receptor gamma, RORγt, and Aryl hydrocarbon receptor, AhR; IL-22 and IL-17) [30,31,32,33,34].

NK cell development requires specific cytokines, such as stem cell factor (SCF), Fms-like Tyrosine kinase receptor 3- Ligand (FlT3-L), IL-7, IL-15, IL-21 and TF including PU.1, ETS Proto-Oncogene 1 (Ets-1), Thymocyte Selection Associated High Mobility Group Box (TOX), Nuclear Factor Interleukin 3 Regulated (NFIL3) and Promyelocytic leukemia zinc finger protein (PLZF) [12,30,33]. Recently, Holmes et al. have shown that Bcl11b TF promote the classical NK cell development and its high expression in NK cells may be correlated with the heterogenous phenotype of adaptive NK cells [35]. In humans, NK cell differentiation occurs through different stages that are characterized by the expression (or lack of the expression) of peculiar surface markers and TF: CD34^+^CD45RA^+^ CD117^−^CD94/NKG2A^−^ stage CD34^+^CD7^+^CD117^+^CD127^+^CD122^+^CD45RA^+^CD94/NKG2A^−^T-bet^+^ stage II, CD34^−^CD117^+^CD122^+^CD161^+/−^NCRs^+/−^Eomes^+^ stage III, CD117^low/−^CD16^+^NKG2D^+^NCRs^+^CD94/NKG2A^+^T-bet^+^eomes^+^CD56^bright^ stage IV, NKG2D^+^NCRs^+^→NKp80^+^CD94/NKG2A^+/−^CD16^+^KIRs^+/−^eomes^+^CD56^dim^ stage V and NCRs^+^NKG2D^+^CD94/NKG2A^+/−^NKp80^+^CD16^+^KIRs^+^NKG2C^+^CD56^+/−^CD57^+^ stage VI (memory-like NK cells).The achievement of stage IV and stage V of NK cell development requires the expression of T-bet (T-box transcription factor TBX21) and Eomes (Eomesodermin)TF, which positively regulate the production of IFN-γ and lytic granules (perforin and granzymes), respectively [36,37].

### 2.2. NK Cells and Tumors

The importance of NK cells in controlling tumor growth and metastasis has been well described. Indeed, their effect against hematological malignancies have been extensively proven while strong evidences support their potential role in the control of solid tumors [38,39,40,41,42] as well.Their ability to recognize cells expressing poor levels of HLA-I molecules and high levels of stress-related molecules favors their specific activation against neoplastic cells, in a way that is complementary to that of T lymphocytes [43,44]. In 2017, Lopez-Soto et al. described an accurate model by which NK cells may play a key role mainly in the inhibition of tumor generation and metastasis dissemination, assuming that they may exert the immune-surveillance at early stages of the disease [45]. In this context, NK cells have been shown to recognize cancer stem cells (CSC) [46,47]. High numbers of tumor-infiltrating NK cells have been correlated with good prognosis and lower relapse rates in patients affected by HER2-positive and triple negative breast cancer, gastrointestinal stromal tumor (GIST), neuroblastoma, head and neck cancer, lung, prostate cancer and melanoma [48,49,50,51,52,53,54]. Moreover, higher levels of NKp46 expression at the tumor site are associated with better survival [55]. Analysis of the NK cell infiltrate in melanoma patients showed that lesions displaying higher NK-cell numbers were found to correlate with the presence of protective stimulatory DCs in the tumor, patient responsiveness to anti–PD-1 immunotherapy and better overall survival [56].

NK cell activity depends on their ability to be recruited to the tumor nest by chemokines. CD56^bright^ cells display a wide range of chemokine receptors: CCR2 (which binds to CCL2/MCP-1, CCL7/MCP-3, CCL12, CCL13/MCP4), CCR5 (recognizing RANTES, MIP1α, and MIP1β), CXCR3 (recognizing CXCL4, CXCL9/MIG, CXCL10/IP10), and CXCR4 (CXCL12 /SDF1α). Moreover, they also express CCR7 (CCL19 and CCL21), which can address the cells to lymph nodes [57,58]. By contrast, CD56^dim^ NK cells show a limited pattern of chemokine receptor expression, including CXCR1 (recognizing CXCL8), CXCR4, and CX3CR1 (recognizing CX3CL1) [57]. On the whole, the different chemokine receptor profile in the two NK cell subsets reflects the higher ability to infiltrate different peripheral tissues and bone marrow of CD56^bright^ as compared to CD56^dim^ NK cells. This is a key issue, since tumor infiltrating (TI) NK cells often display a CD56^bright^ phenotype and/or a reduced expression of the activating receptors, resulting in a de-potentiated anti-tumor activity. In breast cancer, TI-NK cells are enriched in CD56^bright^ cells, and they exhibit low levels of NKp30, NKG2D, DNAM-1, CD16 and poor cytotoxic potential [59]. In early-stage non–small cell lung cancer (NSCLC), TI-NK cells are mostly CD56^dim^, but express limited amounts of activating receptors and show a low degranulation and cytokine release potential [60]. In general, NK cells present in different solid tumors (including lung, gastric, colorectal, and head and neck cancers) are frequently limited in number and may be located within the stroma, far from the tumor nest, and with few exceptions represented by Renal Cell Carcinoma (RCC) and GIST, which are infiltrated by a significant number of NKp46^+^ cells [61,62,63].

Of note, the altered metabolism in patients with tumor and chronic inflammatory disease may also interfere with NK cell development and functions [64]. The different actors present in the TME may determine the activation or exhaustion status of NK cells, as it occurs for T cells. Indeed, tumor cells, macrophages, Treg, and fibroblasts may directly or indirectly modulate NK cell activity by releasing mediators that influence inflammatory anti-tumor response, but also by directly affecting the NK cell-mediated recognition of malignant cells [65,66,67,68,69]. Several tumor immune escape mechanisms have been discovered: up-regulation of HLA class I molecules in myeloma disease; down-regulation of the NKG2D ligands, such as HLA class I chain-related proteins A and B (MICA/B) and unique long 16 (UL16) binding proteins 1–6 (ULBP1-6) in hematological tumors, and down-regulation of activating NK cell receptors due to the release of suppressor cytokines, including transforming growth factor-β (TGF-β) [68]. In melanoma, both tumor cells and tumor-associated fibroblasts can induce the downregulation of activating receptors from the NK cell surface by cell-to-cell contact and the release of PGE2 [70,71]. In addition, it has been demonstrated that indoleamine 2, 3-dioxygenase (IDO) can reduce NK cell cytotoxicity in melanoma patients [70]. The shedding from the tumor cells of activating receptor ligands, such as MICA, MICB and ULBPs, represents another powerful escape strategy, as soluble molecules can mask the activating NK cell receptor or even induce their downregulation from NK cell surface, preventing recognition and killing of tumor cells [72,73]. NK cells may also suffer of tumor epigenetic changes (e.g., histone deacetylase, histone methylation) that can modulate tumor transcriptional activity [74]. Alterations in DNA modifying enzymes such as histone deacetylases (HDACs) or microRNAs, involved in epigenetic gene regulation, repress the expression of MICA/B in leukemic cells and of ULPB in epithelial cancer cells, supporting the role of HDAC inhibitors in anti-cancer therapy [75,76,77]. In-vitro studies of NK/tumor cell cross-talk have also shown that the NK/tumor cell crosstalk can give rise to various functional interactions, with possible contrasting effects on the control of tumor progression. Recently, it has been suggested that NK cells could play a role in epithelial-to-mesenchymal transition of melanoma cells, favoring their progression to more malignant stages [78]. Cantoni et al. showed that NK cells could efficiently degranulate upon incubation with stromal-like Wilm’s tumor cells whereas, on the other hand, these cells could inhibit NK cells via IDO and PGE2 production [79]. Finally, TME-associated hypoxia may contribute to partial abrogation of NK cell activity [80,81]. The characterization and knowledge of these tumor-mediated immune escape mechanisms are providing important clues to overcome them and to improve the efficacy of NK cells in innovative anti-tumor therapies.

## 3. Adoptive NK Cell Therapy

As discussed above, the observation that patients’ NK cells could be dysfunctional or unable to reach the tumor nest prompted researchers to investigate the possibility to design adoptive NK cell-based immunotherapy protocols to improve NK cell anti-tumor response [82,83]. First attempts included the use of autologous NK cells in different types of cancer, however, despite the increase of circulating NK cells and the lack of adverse events such as graft versus host disease (GvHD), these transfers failed to provide significant improvement in patients’ clinical outcome, probably due to the inhibition by self-HLA molecules and to the lack of a sustained in-vivo cell activation [84,85,86]. First evidence that the use of allogeneic, rather than autologous, NK cells could represent a preferential option in cancer treatment came from allogeneic HSCT for hematological diseases, in particular in the haploidentical setting [87]. In patients with leukemia undergoing allogeneic HSCT, NK cells are the first lymphoid subset to appear after transplantation, and play a crucial role in controlling host defense against infections and cancer cells before T cells are reconstituted [87,88,89]. In patients with high-risk AML undergoing haplo-HSCT in complete remission (CR), the use of NK-alloreactive donors (according to the KIR/KIR-L mismatch in the GvH direction model) was associated with better event-free survival (EFS) (67% vs. 18%) [90,91,92]. Important evidences were reported also in pediatric patients in which the overall survival rate was very successful in the presence of NK alloreactivity, especially for the subset of patients with high-risk ALL [93].

However, major issues remain: first of all, the persistence of immature poorly cytolytic CD56^bright^ CD16^−^CD94/NKG2A^+^ NK cells during the first 2 months after transplantation, which may greatly compromise the control of leukemia relapse and infections [94,95]. To address this point, novel transplantation protocols are now under evaluation [93]. Another major issue is the limited availability of fully or, at least, partially compatible donors. In this context, UCB has been shown to represent an important source of CD34^+^ HPCs and of NK cells for allogeneic HSCT [96,97,98]. Of note, NK cells have been shown to recover more quickly after UCB transplantation [99,100].

The findings from the HSCT setting for hematological malignancies prompted the design of adaptive NK cell therapies in other types of cancer diseases, suggesting the possibility to isolate NK cells from PB or UCB to treat cancer patients, used alone or in combination with other therapeutic protocols [83]. In the last two decades, several preclinical and clinical experimentations of NK cell-based cancer immunotherapies have been applied, aimed at evaluating their efficacy [5,6].

## 4. The Challenge of Designing UCB-Derived NK Cell Adoptive Therapy

**Figure 1:**
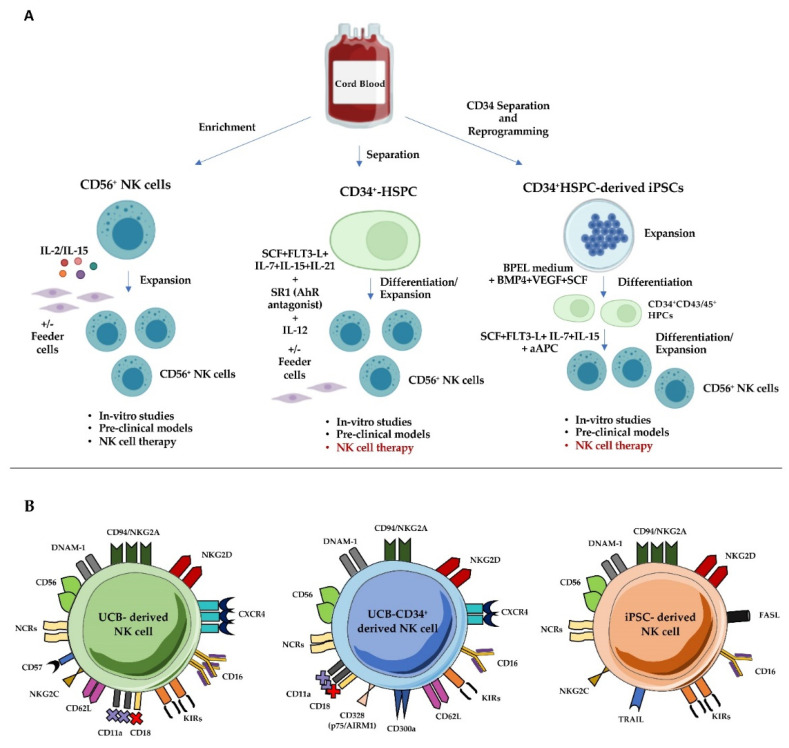
UCB as a source of NK cells. Panel (**A**). Overview of methods to generate, enrich and expand NK cells from UCB. Purified mature NK cells can be obtained directly from UCB and in-vitro expanded in the presence of IL-12 and IL-15 and feeder cells (e.g., K562, 721.221 cell lines). NK cells can also be differentiated from CD34^+^ HSPCs, previously separated from UCB, using appropriate cytokine cocktails including SCF, FLT-3L, IL-7, IL-15, IL-21, StemRegenin-1(SR1) antagonist and IL-12 added at a later time. In some instances, feeder cells can also be used (e.g., OP9 cell line). Finally, NK cells can be generated from UCB CD34^+^ HSPC-derived iPSC. iPSCs are firstly induced to differentiate towards CD34^+^ CD43^+^/CD45^+^ HPCs by using BPEL medium and BMP4, VEGF and SCF factors. HPCs are then differentiated towards NK cells with SCF, FLT-3L, IL-7 and IL-15 cytokines and further expanded with aAPCs. All UCB-derived CD56^+^NK cells are used for in-vitro studies and in preclinical models while at present, UCB-derived mature NK cells are the most exploited source for adoptive cell therapy against hematological and solid tumors. In the HSCT setting, CD34^+^HSC are responsible for the in-vivo generation of NK cells. Panel (**B**). Comparison of UCB-derived NK cells phenotype. Left: UCB-derived mature NK cells; center: CD34^+^HSPC-derived NK cells; right: iPSC-derived NK cells. All types of UCB-derived NK cell express the same levels of CD56, NCRs (NKp46, NKp44 and NKp30), NKG2D and DNAM-1 molecules but UCB-mature NK cells and iPSC-derived NK cells show higher expression of NKG2A and KIRs as compared to UCB-CD34^+^ derived NK cells. UCB-CD34^+^ derived-and iPSC-derived-NK cells express lower amounts of CD16 receptor as compared to UCB-derived mature NK cells. Notably, the expression of memory marker NKG2C on UCB-derived mature NK cells and on iPSC-derived NK cells may activate NK cell degranulation and cytokine production. UCB-derived mature NK cells and UCB-CD34^+^ derived NK cells exhibit higher levels of adhesion molecules such as the heterodimer CD11a/CD18, (LFA-1), and CD62L as compared to iPSC-derived NK cells. The expression of CXCR4 receptor on UCB-CD34^+^ derived NK cell and on UCB-derived mature NK cell could be crucial for homing in the bone marrow. Finally, the expression of TRAIL and FASL on iPSC-derived NK cell could have a pivotal role to activate tumor cell apoptosis. Illustration also created using BioRender.com (accessed on date: 9 August 2022).

UCB is a feasible and suitable source for adaptive immune cell therapy: it can be easily collected and frozen to obtain either mature lymphocytes, or HSC from which it is possible to generate both CD34^+^-HSPC-derived NK cells and iPSC-derived NK cells (Figure 1A). In particular, it has been taken into consideration as a source of mature allogeneic NK cells to be used unmanipulated or after in-vitro expansion and activation [8,9]. However, several challenges must still be faced and their possible resolutions are under evaluation: they are discussed below and summarized in Table 1.

### 4.1. UCB-Derived Mature NK Cells

The percentages of CD3^−^ CD56^+^ cells are similar to those of the PB counterpart, however, a higher proportion of CD56^bright^ cells in UCB compared to PB has been reported. UCB-derived NK cells are more prone to proliferation than PB NK cells [101] and have a high ability to produce IFN-γ when stimulated with IL-12 and IL-18 and high cytotoxicity when stimulated with IL-12 and/or IL-15 [102]. In addition, UCB-derived NK cells express significant levels of CXCR4 that support their capability to home to the BM [103] (Figure 1B).

A limitation of the use of UCB-derived NK cells is represented by the low number of CD56^+^ NK cells that can be recovered. Indeed, the concentration of mononucleated leucocytes is similar in PB and UCB (mean of 1.3 × 10^6^ cells/mL with NK cells representing 5–15% of CB lymphocytes), but the amount of blood that can be collected from CB is generally lower than that collected from PB apheresis. Moreover, compared to PB-, CB-NK cells often show lower expression of CD16, KIRs and DNAM-1 and higher expression of CD94/NKG2A receptors [103]. In particular, the high expression levels of this latter inhibitory immune checkpoint receptor and the low levels of granzyme B may limit the NK cell capability of killing target cells [104] (Figure 1B). In addition, CB-NK cells also show lower expression of homing and adhesion molecules including CD62L, CD54, CD2, and of the heterodimer CD11a/CD18, also known as the Lymphocyte Function-associated Antigen 1 (LFA-1); thus, the necessity of in-vitro cell activation has been taken into consideration to increase their expression [105]. Of note, once activated, UCB-NK cells display functional capabilities similar to those of PB NK cells. Thus, to overcome these issues, several approaches have been evaluated to isolate and activate them in-vitro. The in-vitro expansion of NK cells have shown to benefit from the use of multiple cytokines, including IL-2, IL-12 and IL-18 [101,103,106,107,108]; an important role is exerted by IL-15, which is fundamental during NK cell development and is an NK cell homeostatic mediator [109]. The use of feeder cells, such as K562 and Epstein-Barr virus, transformed B cell lines, which could act as promoters of expanding cells, have been shown to improve NK cell expansion. However, this may represent a limitation in clinical settings because it is mandatory to avoid cell line proliferation [110,111].

Preclinical studies have shown that UCB-derived, in-vitro expanded NK cells displayed good anti-tumor performances as compared to PB NK cells as well. They efficiently produced IFN-γ, TNF-α and degranulated against primary breast cancer or cervical tumor cells [112,113]. As discussed before, the high percentages of CD94/NKG2A^+^ UCB-NK cells could represent a major issue in the limitation of their anti-tumor response, however, the monoclonal antibody (mAb) Monalizumab designed to block NKG2A inhibitory function could enhance NK cell activity [114,115]. Several clinical trials are ongoing for the treatment of hematological and solid tumors; it has been shown that in patients affected by chronic lymphocytic leukemia, the blocking of NKG2A on NK cells with Monalizumab could restore the capability of tumor infiltrating NK cells to exert cytotoxic activity against HLA-E^+^ leukemic cells [115]. Another possibility to enhance NK cell activation is to improve the expression of the activating NK cell receptors and the efficacy of their signaling pathway. NCRs and NKG2D are well-expressed on UCB-derived NK cells, suggesting a possible activation against target cells [103]. In particular, NKG2D can be strongly upregulated upon in-vitro cell activation and is able to mediate NK cell activity against several types of tumor cells [102,116,117]. As for T lymphocytes, the Chimeric Antigen Receptor (CAR) technique has been explored in UCB-NK cells as well. Of note, Herrera et al. showed that UCB-derived CD19-CAR- NK cells, obtained by transducing a CD19-CAR plasmid into IL-2- and IL-15-activated NK cells, displayed higher cell degranulation capability against CD19^+^ cells as compared to unmodified NK cells [118]. In this context, there are two other clinical trials ongoing on CAR-NK cells: a phase 1 study that evaluates NKG2D-CAR-NK cells derived from UCB in patients with relapsed/refractory AML, and a phase 1/2 study of CAR.70-IL-15 transduced UCB-derived NK cells for the management of hematological malignancies (NCT05247957 and NCT05092451, see Table 2).

Recently, novel molecules aimed at specifically redirecting NK cell killing to neoplastic cells have been produced. They are called NK Cell Engagers (NKCE) and would promote the formation of the immunological synapse and thus, of NK cell activation [119]. These molecules are composed by two single chain variable fragments that engage CD16a and specific target tumor antigens and are called bi-specific killer engagers (BIKEs) [119]. It has been also reported that bi-specific antibody CS1-NKG2D favored immune synapse between CS1^+^-Multiple Myeloma and cytotoxic NKG2D^+^-NK cells [120]. More recently, tri-specific killer engagers (TRIKE)-NKCE engagers have been produced, able to trigger CD16, NKp46 or NKp30 NK cell receptors and target CD19 or CD20, to induce killing of pediatric acute B cell Leukemia. Colomar-Carando and colleagues have shown that the use of NKp46-and NKp30-NKCE, incubated with NK cells derived from healthy donors, could potentiate NK cell killing against pediatric B leukemia cell lines and also against ex-vivo isolated primary B cell leukemic blasts. Similarly, the effect was observed by using NK cells derived from HSCT-transplanted patients [121]. Thus, this novel technique could represent a powerful tool also to enhance UCB-derived NK cell anti-leukemic activity, also considering that UCB is used in pediatric HSCT more often [94].

In the clinical setting, UCB-NK cells are actually studied and exploited to improve the outcome of several hematological malignancies (Table 2).

The role of UCB-derived NK cells in an HSCT setting has been well demonstrated [97,98,122]: the mismatch between KIR receptors expressed by donor NK cells and HLA ligands on host cells triggered NK cell cytotoxicity against leukemic cells in patients upon T cell depletion [97,98]. Besides the effects mediated by NK cells expanded in-vivo after UCB-transplantation, the potential role of ex-vivo expanded UCB-derived NK cells is also under evaluation [123]. In the study by Shah et al., UCB-NK cells were infused five days before autologous transplant in patients affected by multiple myeloma (MM), achieving a good partial remission without GHVD reaction. However, four patients relapsed and two of them died. Moreover, UCB-NK cell detectability did not exceed 26 days. Thus, the duration of infused NK cells represents a key issue; as previously discussed, the discovery of the role of IL-15 as NK cell homeostatic mediator highlighted the importance of expanding NK cells more effectively, and of reducing tumor cell activity in patients affected by hematological and non-hematological malignancies [109].

In this context, UCB-NK cells have been shown to be a good source for the generation of CAR-NK cells. Phase 1 and 2 clinical trials reported results obtained using CAR-UCB-NK cells in which the CAR construct was composed of anti-CD19, CD28, CD3ζ IL-15 and inducible caspase 9. Treated patients were affected by CD19^+^ lymphoid tumors and demonstrated a rapid response. Importantly, CAR-NK cells persisted at low levels for at least one year and no significant side effects were observed except for a transient myelotoxicity. Of note, CAR-NK cells could also be detected in patients who did not achieve any response or relapsed, suggesting their possible exhaustion [124,125].

Currently, the efficacy of UCB-derived NK cell therapies in solid tumors is also under evaluation and several clinical trials are ongoing (see the Table 2).

### 4.2. UCB-CD34^+^ Cell-Derived NK Cells

UCB is an important source of CD34^+^ HSPCs to obtain NK cells. Several in-vitro models of NK cell differentiation from CD34^+^HSPCs have been optimized and all of them have represented a powerful tool for several purposes. First, they have provided a unique opportunity to understand the mechanisms sustaining and regulating NK cell development [33,36]; second, they give hints to evaluate the UCB CD34^+^-derived NK cell therapeutic potential in preclinical analyses [126,127,128], and third, they provide the basis for the “off the shelf” NK cell generation for adoptive immunotherapy [8,9,129].

All in-vitro models for NK cell differentiation include the use of an SCF, FlT3-L, IL-7 and IL-15 cytokine cocktail in the medium, in combination with other cytokines such as IL-21 or IL-12, which may promote terminal differentiation by accelerating the acquisition of KIRs, cytokine production and of full cytolytic potential. The addition of other soluble factors (TPO, G-CSF, IL-6) may further support precursor proliferation from fresh or frozen UCB samples (Figure 1A) [128,130,131]. Feeder cells, such as the OP9 cell line, have been shown to promote the generation of large numbers of mature and functional NK cells [132] but, as for the expansion of mature PB-or UCB-NK cells, the use of supportive cell lines may present limitations in the clinical setting. In 2012, two methods have been shown to obtain large numbers of NK cells from UCB-derived CD34^+^ HSPCs: the first approach used the embryonic liver cell line EL08.1D2 in the presence of the above described cytokines, plus IL-3, that was added only at the beginning of the culture [133]. The second protocol is a GMP-compliant system that concerns the using of Glycostem^®^ expansion medium (Oss, The Netherlands) plus addition of several factors such as TPO, GM-CSF, IL-6 and G-CSF added at different time culture intervals [129,131]. This production process occurs in closed, large-scale bioreactors, representing a valuable tool for the generation of clinical grade NK cells for adoptive transfer. Of note, frozen UCB samples have also been shown to support the generation of great numbers of functional UCB CD34^+^-derived NK cells, further underlining the advantages of this source for the generation of NK cells [131,134].

Since the first experiments, in-vitro differentiation models provided unique information on the kinetics of NK cell development which paralleled the results from ex-vivo analyses, in particular those obtained in the allogeneic transplantation setting. Of note, based on these experiments, it has also been stated that UCB-CD34^+^ precursors can give rise to both NK cells and to ILC3. ILC3, which can be identified by the expression of RORγt TF and by the ability to produce IL-22, are generated from CD34^+^ ILC3-specific precursors [135,136,137,138]. Regarding NK cells, during precursor development, the CD161^+^CD56^+^CD117^−^CD7^+^ cells acquire the expression of CD94/NKG2A, NKp80 and LFA-1 and of NCRs, cytolytic granules and the ability to produce IFN-γ. A subset of these NK cells further acquires the expression of KIRs and CD16, witnessing the acquisition of final terminal differentiation (stage IV–V) [33,37]. On the other hand, the lack of expression of CD94/NKG2A and LFA-1 identifies a heterogeneous cell subset (characterized by the CD161^+^CD56^+^CD117^+^CD7^−^LFA-1^−^CD94/NKG2A^−^ phenotype) that may contain both stage III NK cell precursors and ILC3 [135,136,139]. In this model, it is also possible to obtain limited amounts of CD33^+^CD14^+/−^ myelomonocytic cells and of CD83^+^CD86^+^ DCs (authors’ observation), and it has been suggested that, in the presence of hydrocortisone, it is possible to observe NK cell differentiation from myeloid precursors [140]. More recently, an in-vitro platform able to generate all the lineages of ILCs by the use of different combinations of cytokines and feeder cells has been proposed. In particular, it has been shown that the simultaneous presence of SCF, FlT3-L, IL-7, IL-15 and OP9-DL1 feeder cells favored most exclusively the generation of ILC1/NK cells. These results confirm the key role of IL-15 in the generation of NK cells but also underline the role of the NOCTH signaling pathway and provide novel insights into mechanisms that may regulate ILC differentiation and may promote the expansion of different ILC subsets for clinical purposes [141].

Taken together, all the above described in-vitro models indicate a high plasticity of human UCB CD34^+^ precursors, whose cell differentiation could largely depend on microenvironmental stimuli. Thus, studies on UCB precursors may be of great help to understand whether tumors cells, soluble factors or drugs could affect the NK cell developmental process, allowing for performing different types of in-vitro and preclinical analyses. In this context, we showed that IL-1β-releasing AML blasts can interfere with ILC3 and NK cell development [142]. The role of leukemic cells, in particular of the minimal residual disease (MRD), in the modulation of normal hematopoiesis and generation of competent anti-tumor effector cells is a key issue that should also be considered. Indeed, the role of NK cells in leukemic immunosurveillance have been suggested not only for acute diseases but also for chronic myeloid leukemia [143,144]. In particular, a role of NK cells has been suggested in patients undergoing therapy with tyrosine kinase inhibitors (TKI) who reached a deep molecular response (DMR), and were eligible for therapy discontinuation, remaining free of relapse for several years [145,146,147]. However, we observed that the in-vitro administration of these compounds (Imatinib, Nilotinib and Dasatinib) could affect in-vitro myeloid differentiation and also NK cell recovery from UCB CD34^+^ precursors. In particular, Dasatinib seemed to favor the expansion of ILC3 rather than that of mature NK cells [148].

Similarly, the recent increasing clinical use of epigenetic drugs targeting myeloid and solid malignancies prompted researchers to also better evaluate the effect of these compounds on the generation and function of potential anti-tumor effector lymphocytes such as T and NK cells [74,149]. Of note, the addition of EZH1/2 inhibitors to the culture of UCB-CD34^+^ cells undergoing NK cell differentiation favored lineage commitment towards ILC3 differentiation [150]. In our opinion, these results suggest that ex-vivo monitoring of NK cell repertoire should be recommended in patients undergoing these therapeutic protocols, particularly in view to enforce patient NK cell response against tumors. UCB-CD34^+^-derived NK cells display similar functional properties of PB NK cells in terms of IFN-γ, TNF-α production and cytolytic activity. Of note, they express high amounts of HLA-E-specific CD94/NKG2A inhibitory receptor, whereas the percentages of KIR^+^CD56^+^ cells are limited (Figure 1B) [33]. This would suggest that the expression of non-classical HLA-E class I molecules could play a key role in protecting potential tumor targets.

Preclinical studies allow the evaluation of UCB CD34^+^-derived NK cell efficacy in different tumor models and of the possibility of its modulation by putative factors. In this context, the group of Dolstra reported several studies showing how the combined use of IL-2 and IL-15 or the addition of Decitabine epigenetic drug could improve the anti-leukemic activity of NK cells generated from UCB CD34^+^ precursors [127,151]. In particular, they showed that the treatment with hypomethylating agent Decitabine had a positive effect on UCB CD34^+^-derived NK cell anti-tumor response against human AML blasts in-vitro and in-vivo, in NOD/SCID/IL-2Rγ null mice inoculated with human AML leukemia [151]. Of note, in another report, they showed an increased killing by UCB CD34^+^-derived NK cells in combination with gemcitabine in a murine model of ovarian cancer [152]. Thus, UCB CD34^+^-derived NK cells may also display a potential activity against solid malignancies. Hoogstad-van Evert et al. evaluated the potential role of activated UCB CD34^+^-derived NK cells in infiltrating and killing human ovarian cancer spheroids, using an in-vivo-like model. This model concerns the use of a three-cytokine cocktail during in-vitro NK cell differentiation and the addition of the AhR antagonist to prevent ILC3 differentiation [32,128]. Their results support the notion that UCB-CD34^+^-derived NK cells may control tumor cells and also kill cancer stem cells involved in tumor progression and metastasis formation. Of note, the use of IL-15 super agonist N-803 improved the functional activity of CD34^+^-derived NK cells in leukemia and ovarian cancer models both in-vitro and in-vivo, in OC-bearing immunodeficient mice [153]. Currently, in our laboratory, we are evaluating whether UBC-CD34^+^-derived NK cells may infiltrate and kill human NSCLC spheroids, in view to design potential models for the treatment of NSCLC–induced bone metastases.

It must be underlined that only few groups have focused on developing cell therapy approaches based on the in-vitro differentiation of NK cells from UCB CD34^+^ HSCs. These NK cells have been shown to be safe and their use is feasible when considered in the context of allogeneic HSCT, and in a phase I study with patients affected by recurrent ovarian carcinoma [154,155]. In 2017, a clinical trial indicated that NK cells expanded from UCB CD34^+^ HSC and NK progenitor cells could achieve a successful adoptive transfer in elderly AML patients from partially HLA-matched UCB units [156]. Without any cytokine boosting, NK cells persisted in a patient’s PB until day 8 and, of note, NK cell precursor maturation was observed as witnessed by the acquisition of CD16 and KIR receptors. These patients were in complete morphologic remission post treatment with no GVHD and NK cell infusion-related toxicities. Of note, two out of four patients with MRD in BM before NK cell infusion became MRD-negative, and the remission lasted for 6 months, suggesting that UCB CD34^+^-derived NK cells might have contributed to this result.

Recently, a novel clinical trial (NCT04347616) has been approved, in which refractory and relapsed adult AML patients will be eligible to be treated with ex-vivo expanded NK cells derived from allogeneic UCB CD34^+^ HSCs (see Table 1).

### 4.3. UCB-Derived iPSCs as a Source of NK Cells

UCB can also be a source of human iPSCs, which represent an alternative to produce NK cells in a standardized manner for anticancer immunotherapy [157]. Indeed, iPSCs can be obtained through the genetic reprogramming of various somatic cell types by induced overexpression of different stemness transcription factors, usually represented by Oct, Sox2, Klf4, and c-Myc [158]. In addition, UCB-derived purified CD34^+^ HSPC can be easily reprogrammed through previous cell stimulation by SCF, IL-3, and GM-CSF, followed by introduction of the exogenous stemness genes (Figure 1A).

The employment of mature NK cells, either directly isolated from UCB or ex-vivo differentiated from UCB-derived CD34^+^HSPC, is challenging because this approach requires the continuous availability of suitable UCB units and adequate cell numbers to be infused. In this context, iPSCs would represent an appealing solution. Indeed, they can be indefinitely expanded from selected iPSCs clones devoid of genomic alterations in order to ensure a therapeutically safe cell product. Thus, the generation of functionally mature NK cells could rely on a stable, unlimited source of selected and characterized cells.

The differentiation protocol for the development of mature, functional NK cells from UCB-iPSC involves a step-by-step culture method in which iPSC are firstly induced to differentiate towards CD34^+^ CD43^+^/CD45^+^ hematopoietic progenitor cells that, by mimicking embryonal hematopoiesis, originate from the previous stage of hemogenic endothelium [159]. This step involves the formation of spin embryoid bodies that are cultured in a BPEL (bovin serum albumin, polyvinyl alcohol, essential lipids) medium containing BMP4, VEGF, and SCF. The hematopoietic cell precursors are then stimulated to obtain fully differentiated NK cells by the addition of a cytokine cocktail consisting of SCF, FLT3L, IL-3, IL-7, and IL-15, which promotes HPC commitment to the lymphoid lineage and the differentiation and maturation of NK lymphocytes. Further expansion of NK cells can be obtained with IL-2 or with the so-called artificial antigen presenting cells (aAPCs), represented by the NK-sensitive K562 cells that have been genetically modified to express the membrane-bound IL-21 and 4-1BB ligand [160]. Notably, the differentiation protocol has evolved, according to the need of obtaining a serum- and stroma-free cell product suitable for therapeutic infusion [159].

UCB-iPSC-derived NK (iNK) cells are CD3^−^CD56^+^ cells, expressing the most relevant NK cell receptors, including both activating, as CD16, the NCRs, e.g., NKp46 and NKp44 molecules, NKG2D, and inhibitory receptors, as NKG2A and KIRs (Figure 1B). Moreover, cells are fully functional as they show cytotoxic activity against different tumor cell targets both in-vitro and in-vivo [161], mediated by perforins and granzymes of lytic granules and by FasL and TRAIL. In addition, they release IFN-γ and TNF-α upon interaction with target cells. Both phenotype and effector functions of iNK cells are comparable with those shown by conventional NK cell populations [161].Thus, iNK cells represent a suitable alternative tool for immunotherapy approaches employing NK lymphocytes.

Many studies have recently focused on the use of genetic modification to improve NK cell functions and persistence in-vivo. In this context, iNK may represent an advantageous platform as, in this case, cell engineering would be performed on iPSCs, and the starting cell population can be indefinitely expanded and stored as a ready-to-use product for NK cell generation. Following this approach, the sustained stimulation of the IL-15 signaling pathway (to promote cell expansion and activity) has been induced on iNK cells by deleting the CISH gene coding CIS protein, a negative regulator of IL-15 signaling. Alternative strategies have employed an IL-15 superagonist/IL-15Rα fusion construct, activating cytokines or aAPC [162,163]. The potentiation of the NK cell effector function has also been achieved through sustained ADCC by introducing the gene coding for the F158V variant of the CD16 receptor, which is resistant to ADAM17 metalloprotease cleavage [164].

In addition, concerning strategies aimed at increasing NK cell expansion and effector functions, experimental attempts to specifically redirect cells against tumor antigens have been tried. For example, UCB CD34^+^ cell-derived iPSCs have been genetically engineered with CAR constructs. In an ovarian cancer xenograft model, the iNK cells, engineered to target the tumor associated antigen mesothelin, have shown efficient anti-cancer activity and improved survival [165]. A more recent approach has been the use of an engager molecules, represented by an anti-NKG2C/IL-15/anti-CD33 killer engager to specifically target CD33^+^ acute myeloid leukemia cells [166].

Very recently, since 2019, phase I clinical trials have been started by Fate Therapeutics using iPSC-derived NK cells for the treatment of solid or hematologic tumors. In the NCT03841110 trial, the iNK cells are used in combination with checkpoint inhibitor immunotherapy against various advanced solid tumors, whereas, in the NCT04023071 trial, genetically modified iNK cells, expressing the high affinity, cleavage-resistant form of the CD16 receptor, are being employed against hematological malignancies (Table 2). Preliminary clinical data account for the safety and potential efficacy of these biological therapies, thus appearing as a promising alternative to more conventional but less versatile therapeutic products.

## 5. Conclusions

In recent years, the NK cell-based adoptive therapy has emerged as a promising and effective tool to fight cancer. The major source of NK cells has been typically represented by peripheral blood, but some issues on the selection of the HLA-mismatched NK cell subset and on the in vivo persistency of mature PB-NK cells are pushing towards new strategies of effective NK cell supplies. BM has been studied as a source of CD34^+^ HSC rather than for its content of mature NK cells, and, in clinics, it has been successfully utilized in HSCT. In this setting, HSC proved crucial to the development in the patient of persistent NK cells, which were effective in the control of certain hematologic malignancies. Given its limited availability, however, BM has rarely been considered for the preparation of precursor-derived NK cells for therapy. On the other hand, UCB-derived NK cells may represent an important resource to be employed in immunotherapy protocols. In particular, UCB cells can be easily achievable and are taken into consideration as a third-party platform because of the large availability of stored and fresh UCB units. Of note, the existence of Cord Blood Banks may also ensure the selection of donors with peculiar HLA haplotypes. This selection is required for the generation of alloreactive NK cells (e.g., expressing inhibitory KIRs missing recognition of the host HLA-I alleles), which may be more effective in targeting host tumor cells.

UCB mature NK cells can be expanded and activated in-vitro for clinical purposes. Several efforts have been made to improve their potential therapeutic efficacy in terms of proliferation, cytolytic potential, tumor specificity and in-vivo long surviving. In this context, the use of specific cytokine cocktails to expand and stimulate UCB-NK cells in-vitro, as well as the generation of CAR-UCB-NK cells, represent effective approaches and several clinical trials have been approved and are ongoing. To prolong the efficacy and persistence of infused NK cells, the support of IL-15 seems mandatory and thus, the transduction of the IL-15 coding gene should be considered. In this context, iNK cells represent an important platform.

Although UCB-NK cells have been shown to be a reliable tool in hematological malignancies, their use in solid tumors still represents a challenge. The necessity to overcome the inhibitory effects of TME, and to drive CD56^dim^CD16^+^ cytotoxic NK cells into the tumor nest by promoting their recruitment, represents a key issue not only for UCB-NK cells. In this context, the use of the NKCE (BIKE/TRIKE) as well could offer novel opportunities.

Of note, UCB-CD34^+^ cells or UCB-iPSC as sources to expand functional NK cells are still poorly exploited in clinics. However, the availability of feasible GMP protocols should prompt more researchers and clinicians to improve the design of novel trials with these kind of NK cell products. Indeed, the advances in the preparation techniques of NK cells from iPSC, and the improved knowledge of the field, have led to the start of preliminary trials. Nevertheless, some issues regarding NK cell generation and safety still remain, and could have contributed somehow to limit their actual exploitation. Thus, investigations and efforts are required in the next years to make the use of UCB-derived NK cells a common “off the shelf” immunotherapeutic tool. Importantly, as for other novel protocols, the use of UCB-derived NK cells in combination with other therapeutic approaches (chemotherapy, monoclonal antibodies, epigenetic drugs) should be carefully investigated to find out strategies that could significantly improve the efficacy of current treatments, providing a great benefit for cancer patients.

## Figures and Tables

**Table 1:** The challenge of designing UCB-derived NK cell adoptive therapy.

Challenges	Possible Improvements/Resolutions
**Low numbers of NK cells**	Purification and in-vitro expansion of mature NK cells with IL-2/IL-15 ± irradiated feeder cells Exploitation of approved GMP protocols for large scale generation of NK cells from UCB CD34^+^ HSPC Generation of UCB-iPSC-derived NK cells (iNK)
**Low expression of activating receptors, adhesion molecules and Granzyme B**	In-vitro activation with IL-15 plus IL-12/IL-18
**In-vitro survival and sustained activation of infused NK cells**	IL-15 administration Generation of UCB-CAR NK cells equipped with IL-15 construct IL-15 superagonist/IL-15Rα fusion construct in iNK Deleting in iNK cells the CISH gene coding for CIS protein, a negative regulator of IL-15 signaling
**Improvement of NK cell homing in tumor nest and of their antitumor activity**	Generation of UCB CAR NK cells equipped with CD16/NKG2D/chemokine receptors/tumor specific antigens Generation of BIKE/TRIKE molecules Use of anti-CD94/NKG2A antibodies

**Table 2:** Ongoing clinical trials with UCB-NK cell-based therapies in hematological and solid tumors. HCC (Hepatocellular Carcinoma); RCC (Renal Cell Carcinoma);ALL (Acute Lymphoid Leukemia); AML (acute myeloid leukemia); Rec.(Recurrent); Rec. Mal. (Recurrent Malignant); CML (Chronic Myeloid Leukemia); CLL (Chronic Lymphoid Leukemia); CRC (Colorectal Cancer); MDS (Myeloid dysplastic syndrome); Acc.(Accelerated); HSCT (Hematopoietic stem cell transplant).

Trials	NCT Number	Disease	Trial Phase	Interventions
1	NCT05110742	Hematological Malignancy	Phase 1 Phase 2	Drug: Fludarabine Phosphate Cyclophosphamide Biological:CAR.5/IL15-UCB-NK cells
2	NCT01914263	HCC RCC Lung Cancer	Phase 1	Biological: cytokine induced NK cells
3	NCT03056339	B-Lymphoid Malignancies ALL CLL Non-Hodgkin’s Lymphoma	Phase 1 Phase 2	Drug: Fludarabine Drug: Cyclophosphamide Drug: Mesna Biological: iC9/CAR.19/IL15-UCB-NK Drug: AP1903
4	NCT01729091	Plasma Cell Leukemia Plasma Cell Myeloma	Phase 2	Autologous HSCT Biological: Elotuzumab Other: Laboratory Biomarker Analysis Drug: Lenalidomide Drug: Melphalan Biological: NK Cell Therapy Biological: UCB-Derived Lymphocyte
5	NCT03420963	Rec.Cutaneous Melanoma Rec.Lip and Oral Cavity Carcinoma Rec.Malignant Endocrine Neoplasm Rec. Mal.Female and Male Reproductive System Neoplasm	Phase 1	Biological: UCB-derived Expanded Allogeneic NK Cells Drug: Cyclophosphamide Drug: Etoposide
6	NCT04796675	ALL CLL Non Hodgkin’s Lymphoma	Phase 1	Drug+Biological: Fludarabine +Cyclophosphamide + UCB CAR-NK-CD19 Cells
7	NCT03841110	Advanced Solid Tumors Lymphoma Gastric Cancer CRC Head and Neck Cancer Squamous Cell Carcinoma EGFR+Solid Tumor HER2+ Breast Cancer HCC Small Cell Lung Cancer	Phase 1	FT500 (iPSC-derived NK cell product) Drug: Nivolumab Drug: Pembrolizumab Drug: Atezolizumab Drug: Cyclophosphamide Drug: Fludarabine Drug: IL-2
8	NCT01619761	Acc. Phase CML, BCR-ABL1 + Acute Biphenotypic Leukemia ALL in Remission	Phase 1	Allogeneic HSCT Drug: Cyclophosphamide Drug: Fludarabine Phosphate
9	NCT04347616	AML refractory AML relapsed adult	Phase 2	Biological: UCB-CD34^+^ NK cells Drug: IL-2
10	NCT04023071	AML B-cell lymphoma	Phase 1	FT516 (iPSC-derived NK cell product) Drug: Rituximab or Obinutuzumab
11	NCT05247957	AML refractory AML relapsed adult	Phase 1	Biological: UCB NKG2D-CAR-NK cells
12	NCT05092451	AML B-cell lymphoma MDS	Phase 1 Phase 2	Drug: Cyclophosphamide Biological: CAR.70/IL-15- UCB-NK cells Drug: Fludarabine Phosphate
